# Anhedonia as a transdiagnostic symptom across psychological disorders: a network approach

**DOI:** 10.1017/S0033291722000575

**Published:** 2023-07

**Authors:** Melissa G. Guineau, N. Ikani, M. Rinck, R. M. Collard, P. van Eijndhoven, I. Tendolkar, A. H. Schene, E. S. Becker, J. N. Vrijsen

**Affiliations:** 1 Behavioural Science Institute, Radboud University, Nijmegen, The Netherlands; 2 Overwaal, Center of Expertise for Anxiety, Obsessive-Compulsive, and Posttraumatic Stress Disorders, Pro Persona, Institute for Integrated Mental Health Care, Nijmegen, The Netherlands; 3 Department of Psychiatry, Radboud University Medical Center, Nijmegen, The Netherlands; 4 Donders Institute for Brain, Cognition and Behaviour, Radboud University Nijmegen, Nijmegen, The Netherlands; 5 Depression Expertise Center, Pro Persona Mental Health Care, Nijmegen, The Netherlands; 6 Department of Psychiatry and Psychotherapy, University Hospital Essen, Essen, Germany

**Keywords:** Anhedonia, comorbidity, network approach, RDoC

## Abstract

**Background:**

Anhedonia is apparent in different mental disorders and is suggested to be related to dysfunctions in the reward system and/or affect regulation. It may hence be a common underlying feature associated with symptom severity of mental disorders.

**Methods:**

We constructed a cross-sectional graphical Least Absolute Shrinkage and Selection Operator (LASSO) network and a relative importance network to estimate the relationships between anhedonia severity and the severity of symptom clusters of major depressive disorder (MDD), anxiety sensitivity (AS), attention-deficit hyperactivity disorder (ADHD), and autism spectrum disorder (ASD) in a sample of Dutch adult psychiatric patients (*N* = 557).

**Results:**

Both these networks revealed anhedonia severity and depression symptom severity as central to the network. Results suggest that anhedonia severity may be predictive of the severity of symptom clusters of MDD, AS, ADHD, and ASD. MDD symptom severity may be predictive of AS and ADHD symptom severity.

**Conclusions:**

The results suggest that anhedonia may serve as a common underlying transdiagnostic psychopathology feature, predictive of the severity of symptom clusters of depression, AS, ADHD, and ASD. Thus, anhedonia may be associated with the high comorbidity between these symptom clusters and disorders. If our results will be replicated in future studies, it is recommended for clinicians to be more vigilant about screening for anhedonia and/or depression severity in individuals diagnosed with an anxiety disorder, ADHD and/or ASD.

Comorbidity between major depressive disorder (MDD), anxiety disorders, attention-deficit hyperactivity disorder (ADHD), and autism spectrum disorder (ASD) is high (Antshel, Zhang-James, Wagner, Ledesma, & Faraone, 2016; Ghaziuddin, Ghaziuddin, & Greden, 2002; Grevet et al., 2006; Hirschfeld, 2001; Joshi et al., 2013; Kaufman & Charney, 2000; Kessler et al., 2006a; Kessler, Merikangas, & Wang, 2006b; Schatz & Rostain, 2006; van Loo, Romeijn, de Jong, & Schroevers, 2013). To illustrate, 67–73% of the patients diagnosed with an anxiety disorder, are also diagnosed with MDD (Kaufman & Charney, 2000; Lamers et al., 2011). Comorbidity rates between ADHD and ASD are estimated between 14% and 78% (Gargaro, Rinehart, Bradshaw, Tonge, & Sheppard, 2011) and comorbidity rates between these two neurodevelopmental disorders, MDD, and anxiety disorders are estimated between 25% and 75% (Grevet et al., 2006; Joshi et al., 2013; Kessler et al., 2006a; Schatz & Rostain, 2006). This pattern of co-occurrence questions the validity of distinct diagnostic categories (Forbes, Tacket, Markon, & Krueger, 2016). Instead, comorbid disorders may represent manifestations of common underlying transdiagnostic psychopathology features that cut across diagnostic boundaries (Cuthbert, 2014; Forbes et al., 2016; Kessler et al., 2011).

The frequent comorbidity among supposedly distinct disorders motivated the National Institute of Mental Health (NIMH) to develop the Research Domain Criteria (RDoC) framework. The goal of RDoC is to explore transdiagnostic, underlying dimensions of psychopathology across a broad range of disorders (Cuthbert, 2014; Insel et al., 2010). The Negative Valence System is one such dimension and includes the subconstruct ‘loss’, which describes a state of deprivation, which may include behavior, status, loved ones or relationships (Cuthbert, 2014). This construct can be assessed at different levels (e.g. molecules, behavior, physiology). One element on the behavioral level is anhedonia, which can be understood as a loss of pleasure in formerly enjoyable activities and includes a loss of effort/motivation, desire, anticipation, and consummatory pleasure (Rizvi, Pizzagalli, Sproule, & Kennedy, 2016). Though traditionally linked to MDD, anhedonia is a common symptom of mental disorders as it is seen in e.g. ADHD, ASD, schizophrenia, substance use disorders, and anxiety disorders [American Psychiatric Association (APA), 2013; Bringmann, Lemmens, Huibers, Borsboom, & Tuerlinckx, 2015; Chevallier, Grèzes, Molesworth, Berthoz, & Happé, 2011; Gard, Kring, Gard, Horan, & Green, 2007; Hatzigiakoumis, Martinotti, Giannantonio, & Janiri, 2011; Kashdan, Zvolensky, & McLeish, 2008; Meinzer, Pettit, Leventhal, & Hill, 2014; Sarramon, Verdoux, Schmitt, & Bourgeois, 1999]. Anhedonia is also a central part of the diagnosis of MDD, ASD, and anxiety disorders (APA, 2013).

Anhedonia is suggested to be related to dysfunctions in the reward system and/or affect regulation (Keedwell, Andrew, Williams, Brammer, & Philips, 2005; Rizvi et al., 2016). To illustrate, MDD patients display deficits in reward responsiveness, reward anticipation and increased suppression of positive and negative affect (Beblo et al., 2012; Pizzagalli, Losifescu, Hallet, Ratner, & Fava, 2008; Vrieze et al., 2014b). Relatedly, decreased processing of rewarding cues in ADHD patients, could give rise to anhedonia (Meinzer et al., 2014). For anxiety patients, reduced acceptance of emotional distress may contribute to anhedonia (Kashdan et al., 2008) and theory-of-mind deficits in ASD make social interactions less pleasurable and rewarding (Krach, Paulus, Bodden, & Kircher, 2010). Anhedonia is seen across these often-comorbid mental disorders and may present a transdiagnostic underlying feature for psychopathology (Keedwell et al., 2005; Rizvi et al., 2016).

Anhedonia might also be linked to symptom severity of mental disorders. Anhedonia correlated positively with ASD symptom severity (Chevallier et al., 2011; Novacek, Gooding, & Pflum, 2016) and interacted with ADHD severity and higher levels of depression severity (Babinski, Waschbusch, & Waxmonsky, 2019). However, results regarding MDD and anxiety are mixed. Several studies demonstrate that anhedonia is predictive of MDD (Loas, Salinas, Guelfi, & Samuel-Lajeunesse, 1992; Rey, Jouvent, & Dubal, 2009; Vrieze et al., 2014a) and anxiety severity (Keedwell et al., 2005; Rey et al., 2009) whereas another study demonstrated that anhedonia did not correlate with MDD severity (e.g. Schrader, 1997) nor anxiety symptoms (Kashdan et al. 2008). On the other hand, anxiety sensitivity (AS), a core feature of anxiety disorders (Mantar, Alkin, & Yemez, 2011; Taylor, 1995, 1996), was found to be associated with more severe anhedonia (Kashdan et al., 2008). Nevertheless, Watson and Naragon-Gainey (2010) note that anhedonia has stronger associations with MDD than with anxiety disorders, but this might be because anhedonia is considered a core characteristic of MDD. Although anhedonia is implicated in a range of mental disorders and is related to poor treatment outcomes (Craske, Meuret, Ritz, Treanor, & Dour, 2016; McIntyre et al., 2015), it's possible transdiagnostic role is underexplored.

A network approach is a promising way of investigating the relationships between anhedonia and validated symptom clusters characterizing depression, AS, ADHD, and ASD symptom severity, because it aims to identify plausible relations among symptom clusters that might be masked by traditional statistical approaches (Costantini et al., 2015). More specifically, network analysis allows for the examination of how symptoms of one disorder (i.e. nodes) are associated with symptoms of another disorder (represented by edges; Borsboom & Cramer, 2013; Cramer, Waldorp, van der Maas, & Borsboom, 2010). In doing so, it is also possible to identify central features of the network that reflect transdiagnostic features that cut across diagnostic boundaries (Cramer et al., 2010).

The current study aims to explore the network structure and centrality of anhedonia severity and symptom severity clusters of depression, AS, ADHD, and ASD in a heterogeneous clinical sample with a high level of comorbidity (81.3%), close to clinical practice (Antshel et al., 2016; McIntosh et al., 2009). We computed a graphical LASSO network (Friedman, Hastie, & Tibshirani, 2008) to begin visualizing associations between symptom clusters, and a relative importance network (Grömping, 2006) to assess the directionality of associations. We first explored the strongest edges within the network and then focused on the nodes' centrality values.

MDD, anxiety disorders, ADHD and ASD are often comorbid, yet mechanisms that potentially underlie this comorbidity remain relatively underexplored. Research that did focus on the negative valence system, has often been limited to specific disorders. For example, anhedonia has thus far predominantly been investigated in the context of depression (e.g. Keedwell et al., 2005; Loas et al., 1992; McIntyre et al., 2015). This study informs us about anhedonia as a transdiagnostic psychopathological feature in symptom clusters of depression, AS, ADHD and ASD, and which may partially explain their high comorbidity rates observed in clinical practice (Zbozinek et al., 2012). Importantly, this study focused on anhedonia in the context of symptom severity clusters instead of diagnostic categories. As such, our study is in line with the goals of the RDoC framework to investigate the mechanisms pertaining to mental health dysfunctions from a dimensional point of view (Cuthbert, 2014; Insel et al., 2010).

## Method

### Participants

This study used data of the ongoing MIND-Set study (Measuring Integrated Novel Dimensions in neurodevelopmental and stress-related mental disorders), conducted at the Department of Psychiatry of the Radboud university medical center (Radboudumc) and the Donders Institute, Nijmegen, The Netherlands. The MIND-Set study (*N* *=* 559 at the time of writing the manuscript) is an observational, cross-sectional study of Dutch adult (age >17 years) patients who were diagnosed with one or more of the following disorders: mood disorders, anxiety disorders, ADHD, or ASD, and possibly a comorbid substance use disorder. The study included mostly outpatients (*N* = 555, 99.6%), but eligible inpatients were also allowed to participate.

The current study excluded two patients as they did not meet the inclusion criteria for the current study (i.e. no diagnosis of mood disorders, anxiety disorders, ADHD or ASD). This resulted in a final analytical sample of 557 patients. This sample included slightly more males (*n* = 302; 54.2%) than females. The mean age was approximately 39 years (s.d. = 14; range 17 to 79). Educational level was reasonably distributed being low for 81 participants (14.5%), medium for 209 participants (37.5%), high for 206 participants (36.9%), unknown for 36 participants (6.5%), and 19 participants (3.4%) did not fulfill any graduation. Within this sample, 232 participants (41.6%) were diagnosed with MDD, 176 participants (31.5%) were diagnosed with an anxiety disorder, 215 participants (38.5%) were diagnosed with ADHD, and 133 participants (23.8%) were diagnosed with ASD. Of the 176 participants with anxiety disorders, 38 participants (21.5%) were diagnosed with panic disorder, 6 participants (3.4%) were diagnosed with agoraphobia, 16 participants (9.0%) were diagnosed with obsessive compulsive disorder (OCD), 48 participants (27.2%) were diagnosed with PTSD, 36 participants (20.4%) were diagnosed with GAD, 50 participants (28.4%) were diagnosed with a social phobia, 15 participants (8.5%) were diagnosed with a specific phobia, and 19 participants (10.7%) were diagnosed with an anxiety disorder not otherwise specified (NOS).^†^^1^ Note that comorbidity in the sample was common (81.3%) and that the sum score of the differential diagnoses exceeds the number of patients in total that were diagnosed with an anxiety disorder. See Table 1 for an overview of sample characteristics.

**Table 1.** Sample descriptives of patients (*N* = 557) diagnosed with MDD, anxiety disorders, comorbid substance use disorders, ADHD or ASD

	MDD (*n* = 232)	Anxiety (*n* = 176)	Substance use (*n* = 152)	ADHD (*n* = 215)	ASD (*n* = 133)
Age *M* (s.d.)	41 (15.0)	39 (13.5)	39 (13.7)	35 (11.6)	35 (12.5)
Sex (% males)	55%	53%	65%	55%	65%
Educational level (%)					
(Almost) no education	4%	6%	3%	3%	2%
Low	17%	16%	16%	13%	13%
Middle	40%	33%	45%	44%	39%
High	36%	38%	28%	31%	43%
Anhedonia *M* (s.d.)	18 (4.5)	18 (4.5)	16 (5.3)	12(5.2)	14 (5.1)
IDS-SR *M* (s.d.)	42 (11.4)	36 (12.5)	36 (13.9)	29 (12.6)	29 (12.4)
ASI *M* (s.d.)	17 (9.9)	19 (9.8)	15 (9.9)	14 (8.3)	15 (9.2)
CAARS-SS *M* (s.d.)	19 (6.0)	18 (5.7)	19 (6.0)	19 (5.5)	18 (6.1)
AQ-50 *M* (s.d.)	124 (20.0)	128 (20.6)	124 (17.4)	119 (20.0)	140 (18.3)
Comorbidity MDD *N* (%)	–	70 (39%)	73 (48%)	51 (23%)	35 (26%)
Comorbidity anxiety *N* (%)	70 (30%)	–	41 (27%)	53 (25%)	41 (31%)
Comorbidity substance use *N* (%)	73 (31%)	41 (23%)	–	70 (33%)	26 (20%)
Comorbidity ADHD *N* (%)	51 (22%)	53 (30%)	70 (46%)	–	44 (33%)
Comorbidity ASD *N* (%)	35 (15%)	41 (23%)	26 (17%)	44 (20%)	–

*Note*. The structured interviews did not provide information about which diagnosis was primary or secondary. Patients with substance use disorders were only included in the study if they were also diagnosed with comorbid MDD, anxiety, ADHD or ASD. This resulted in the exclusion of two patients with a substance use disorder only, leaving a total sample of 557 patients. Educational level was divided into four levels: (almost) no education (elementary education or education not finished), low (lower vocational and general secondary education), middle (intermediate vocational and higher secondary education) and high (higher vocational education and university). Categories of substance use disorders included: nicotine (*n* = 99), alcohol use disorders (*n* = 40), cannabis (*n* = 33), sedatives (*n* = 12), opiates (*n* = 5), stimulants (*n* = 5), cocaine (*n* = 4), gambling (*n* = 2), GHB (*n* = 2) and ecstasy (*n* = 1).

### Procedure

Patients referred to the Department of Psychiatry of the Radboudumc received an invitation letter for their first appointment and a MIND-Set information letter at home. Before the first appointment, all patients filled out standard clinical questionnaires at home via an online secure questionnaire program. These included the symptom severity scales used in the current study. If preferred, a paper copy of the questionnaires was sent to the patients’ home address.

During the clinical diagnostic phase at the psychiatric department, patients were diagnosed in accordance with Dutch guidelines. The Structured Clinical Interview for DSM-IV Axis I Disorders (SCID-IV-RV, referred to as SCID-I; First, Spitzer, Gibbon,& Williams, 1996) was used to diagnose mood and anxiety disorders. The Structured Interview Measurements in the Addictions for Triage and Evaluation (MATE; Schippers & Broekman, 2010) was used to assess substance use disorders. The Diagnostic Interview for ADHD in adults, second edition (DIVA 2.0; Kooij & Francken, 2010) was used to diagnose ADHD, and the Dutch Interview for Autism Spectrum Disorders in Adults (NIDA; Vuijk, 2014) was used to diagnose ASD. Before the diagnostic interview of the DIVA and the NIDA were administered, a clinician explained the patient that it is necessary to administer this interview in the presence of a significant other. The clinician helped the patient in deciding who could join the interview and indicated the preference for a parent/brother/sister joining the interview (i.e. someone who knows the patient since they were a child). Thus, to ensure that clinicians could assess retrospective and hetero-anamnestic information, both the DIVA and the NIDA were always administered in the presence of a significant other. Their response was used in the diagnostic process. All clinicians involved in the clinical diagnostic procedure were experienced psychiatrists and psychologists who received extensive training in clinical interviewing.

Patients who were willing to participate in the MIND-Set study signed an informed consent form at the end of their diagnostic procedure. When patients consented to participation, the structurally collected questionnaire data could be used for research purposes. Also, biological measures (e.g. blood samples, hair samples), neuropsychological tests, and neuro-imaging measures followed. These measures are not part of the current study. The MIND-Set study has been approved by the local medical ethics committee (Commissie Mensgebonden Onderzoek Arnhem – Nijmegen). The authors assert that all procedures contributing to this work comply with the ethical standards of the relevant national and institutional committees on human experimentation and with the Helsinki Declaration of 1975, as revised in 2008.

### Measures

#### Severity of anhedonia

Seven items which represent an anhedonia factor in the Outcome Questionnaire (OQ-45-2;^2^ Lambert et al., 2006), are used to assess the severity of anhedonia (Minami et al., 2009). These seven anhedonia items are combined into the anhedonia factor, ranging from 0–27 (higher scores indicate more severe anhedonia). The items (e.g. ‘I am not interested in anything’ or the reversed item ‘I enjoy my leisure time’) were answered on a 5-point Likert scale, ranging from 0 (*never*) to 4 (*almost always*). The anhedonia factor correlated significantly with the SCID item representing anhedonia according to the DSM-IV, *r*_s_(441) = 0.536, *p* < 0.001. In the current sample, the internal consistency of the factor was good (*α* = 0.87).

#### Depressive symptoms

The Inventory of Depressive Symptomatology Self-Report (IDS-SR; Rush, Gullion, Basco, Jarrett, & Trivedi, 1996) assesses the severity of depressive symptoms in the past seven days. The 30 items (e.g. ‘Feeling sad’) are answered on a four-point scale (0–3) that describes different responses (e.g. with 0 indicating ‘*I do not feel sad*’ and 3 indicating ‘*I feel sad nearly all the time*’). In the current sample, the internal consistency of the IDS-SR was good (*α* = 0.86).

#### Anxiety sensitivity

The Anxiety Sensitivity Index (ASI; Reiss, Peterson, Gursky, McNally, 1986; Rodriguez, Bruce, Pagano, Spencer, & Keller, 2004) assesses AS. As an individual trait concept, AS determines a person's proneness to become frightened by anxiety-related sensations (Reiss, 1997). We selected the ASI as it allows for a transdiagnostic and broad concept of the anxiety domain for both subclinical patients as well as for patients with comorbid anxiety disorders, in a sensitive way. Moreover, previous studies (e.g. Grös, Simms, Antony, & McCabe, 2007; Julian, 2011) have shown that other measures, such as the STAI, have problems in differentiating anxiety from depression symptoms. By using the ASI, we aimed to use a ‘pure’ anxiety measure, without overlapping depressive symptoms. The 16 items (e.g. ‘It scares me when my heart beats rapidly’) are answered on a 5-point Likert scale, ranging from 0 (*barely*) to 4 (*extremely much*). In the current sample, the internal consistency of the ASI was good (*α* = 0.86).

#### ADHD symptoms

The Conners' Adult ADHD Rating Scale, Short Version (CAARS-SS; Connors, Erhardt,& Sparrow, 1999) assesses the presence and severity of ADHD symptoms. The 26 items (e.g. ‘I am looking for fast exciting activities’) are answered on a 4-point Likert scale, ranging from 0 (*not at all/never*) to 3 (*very much/very frequently*). In the current sample, the internal consistency of the CAARS-SS was good (*α* = 0.88).

#### Autistic traits

The 50-item Autism Spectrum Quotient (AQ-50; Baron-Cohen, Wheelwright, Skinner, Martin, & Clubley, 2001) assesses autistic traits. Items (e.g. ‘I notice sounds which other people don't seem to notice’) are answered on a 4-point Likert scale, ranging from 1 (*totally agree*) to 4 (*totally disagree*). In the current sample, the internal consistency of the AQ-50 was excellent (*α* = 0.90). The mean values and standard deviations for all measures are described in Table 2.

**Table 2.** Mean (s.d.) and range for scores on Anhedonia, the IDS-SR, ASI, CAARS-SS and AQ-50

Measurement	*M* (s.d.)	Range	Skewness *M* (s.e.)	Kurtosis *M* (s.e.)
Anhedonia	14.22 (5.57)	0–27	−0.21 (−0.98)	−0.56 (−1.27)
IDS-SR	32.59 (14.18)	2–74	0.08 (−0.38)	−0.45 (−1.03)
ASI	15.16 (9.46)	0–62	0.79 (3.63)	0.83 (1.90)
CAARS-SS	17.57 (6.13)	1–32	−0.23 (−1.08)	−0.40 (−0.92)
AQ-50	123.42 (20.93)	67–191	0.10 (0.48)	−0.19 (−0.44)

IDS-SR, Inventory of Depressive Symptomatology Self-Report; ASI, Anxiety Sensitivity Index; CAARS-SS, Conners' Adult ADHD Rating Scale, Short Version; AQ-50, 50-item Autism Spectrum Quotient. Note that scores on the ASI were positively skewed, this may be due to the high number of males in the sample who tend to underreport their fear and anxiety symptoms (Egloff & Schmukle, 2004; Pierce & Kirkpatrick, 1992).

### Data analyses

All analyses were conducted in the R programming environment (Version 3.6.3; R Core Team, 2020), the R code is available on the Open Science Framework (https://osf.io/6mjdf/). As data collection is still ongoing, the de-identified dataset is available upon request from the first author. We computed two types of networks: a graphical LASSO network to visualize the associations between the symptom clusters and a relative importance network to assess directionality.

#### Graphical LASSO network

The regularized partial correlation network was computed using a graphical Least Absolute Shrinkage and Selection Operator (LASSO), by using the R packages *qgraph* (Epskamp, Cramer, Waldorp, Schmittmann, & Borsboom, 2012) and *glasso* (Friedman et al., 2008). Graphical LASSO first computes regularized partial correlations between all nodes, thereby controlling for the influence of all other nodes in the network. Next, it shrinks small associations to zero. This procedure removes potentially false-positive associations and returns a sparse network compromising only the edges that are likely genuine (Epskamp, 2016; Epskamp & Fried, 2018).

The *qgraph* package implements the graphical LASSO regularization in combination with extended Bayesian information criterion (EBIC) model selection. Graphical LASSO uses a tuning parameter (*γ*) to control the degree to which regularization is applied. This tuning parameter controls the trade-off between including false-positive edges and removing true edges. The tuning parameter is advised to be set between zero and 0.5 (Foygel & Drton, 2010). A tuning parameter close to 0.5 can be conservative and not reflect the true model, whereas a tuning parameter close to zero can be useful in exploratory and hypothesis-generating research (Hevey, 2018). We chose a moderately conservative value of *γ* to 0.25, to increase specificity and interpretability (Epskamp & Fried, 2018).

As ASI scores were positively skewed, we computed an additional graphical LASSO network after performing a non-linear transformation of ASI scores, to test whether skewness was a potential confound.

#### Node centrality for the graphical LASSO network

The most central symptom clusters in the network were identified using betweenness, closeness, and strength indices (Costantini et al., 2015). Betweenness is calculated by summing the number of times that a given node lies on the shortest path between two other nodes. Closeness is calculated as the inverse of all shortest path lengths between the given node and all other nodes in the network. Node strength calculates the direct connection of a given node to all other nodes in the network, by summing all edge weights a node is directly connected to. Each centrality index was computed using the R package *qgraph* (Epskamp, Borsboom, & Fried, 2018). For all centrality measures, higher values reflect stronger associations in the network.

#### Stability analyses

To test whether the results allow for reliable interpretations, we examined the stability of the network using the R package *bootnet* (Epskamp et al., 2018). Edge weight stability was tested using a non-parametric 1000-sample bootstrap technique to calculate 95% confidence intervals (CIs) for the edges. An additional 1000-sample dropping bootstrap was conducted to estimate the stability of the centrality indices. This procedure yields a correlation stability (CS) coefficient. The CS-coefficient reflects the maximum proportion of participants that could be dropped from the analyses, while still retaining a correlation of at least 0.7 with the centrality values estimated in the original network within a 95% CI. The recommended CS-coefficient should be at least 0.25, but preferably above 0.5. Finally, bootstrapped significance tests were used to test for significant differences between edge-weights or node centralities. A bootstrapped CI was constructed around the difference between bootstrap values of edge-weights or node centralities. Edge weights or node centralities differed from one-another when zero was not in the bootstrapped CI (Epskamp et al., 2018).

#### Relative importance network

We then computed a relative importance network by using the R package *relaimpo* (Grömping, 2006). A relative importance network considers the amount of explained variance that each node makes to *R*^2^ after controlling for multicollinearity (Johnson & LeBreton, 2004). These direct effects are adjusted for all other nodes in the network. Each edge is assigned a relative importance metric (*lmg*) between 0 and 1 (Grömping, 2006; Johnson & LeBreton, 2004). Edges in a relative importance network are weighted and directed. This procedure has been shown to be more stable and generalizable compared to other network procedures to assess directionality [i.e. a Bayesian directed acyclic graph (DAG); Forbes, Wright, Markon, & Krueger, 2017).

#### Node centrality for the relative importance network

Similar to the Graphical LASSO network, we also computed centrality indices (Costantini et al., 2015). Within a relative importance network, the strength parameter can be broken up into in-strength and out-strength. In-strength is calculated by summing the directed edge weights originating from other nodes and ending at a given node. It thus quantifies the extent to which a node is influenced by other nodes in the network. In contrast, out-strength is calculated by summing the directed edge weights originating from a given node and ending at all other nodes. It thus quantifies the extent to which a given node exerts predictive influence on other nodes in the network. Again, higher values reflect greater centrality (Epskamp et al., 2018). We also assessed the stability of the relative importance network, similar to the graphical LASSO network.

#### Missing data

Overall, 0.017% of participants had missing data (Anhedonia factor: 2 patients, IDS-SR: 4 patients, AQ-50: 4 patients). There was no missing data on ASI and CAARS-SS items. The network was estimated using complete pairwise observations (i.e. using all available data).

## Results

### Graphical LASSO network

**Fig. 1.**
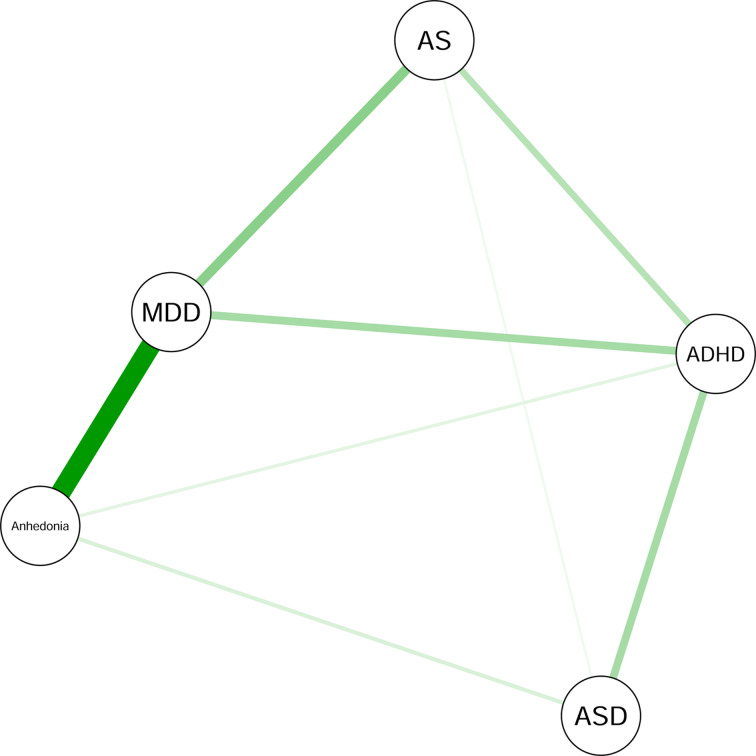
Graphical LASSO network. Nodes represent anhedonia severity or severity of symptom clusters of MDD, AS, ADHD, or ASD. All edges represent positive regularized partial correlations. The thickness of an edge reflects the magnitude of the association (the thickest edge representing a value of 0.61).

Figure 1 depicts the graphical LASSO network, which includes regularized partial correlations and limits spurious associations. Online Supplementary Fig. S1 depicts the network with the transformed measure of AS, this transformation did not change the network structure. Edge weights reflect the strength of associations and edge color the direction (green edges reflect positive associations). For all networks, node placement was determined by Fruchterman and Reingold's (1991) algorithm, which results in a visually appealing graph where nodes generally do not overlap and edges have approximately the same length (Jones, Mair, & McNally, 2018).

Several features are immediately apparent. First, depression symptom severity emerged as a central node, connected to anhedonia, AS, and ADHD symptom severity. Anhedonia severity also emerged as a central node, connected to depression, ADHD, and ASD symptom severity. ADHD symptom severity and AS symptom severity were also connected, as well as AS and ASD symptom severity. The correlation matrix with all the regularized partial correlation coefficients is presented in online Supplementary Table S2. Bootstrapped CI's for the edges indicate that most edges were fairly stable, as demonstrated by relatively narrow CI's (online Supplementary Fig. S2). The bootstrapped significance test (online Supplementary Fig. S3) revealed that the edges between the symptom severity clusters of anhedonia and depression, depression and AS, depression and ADHD, and between ADHD and ASD were significantly stronger than at least half of the other edges in the network.

#### Node centrality

These dynamics are further reflected in the centrality indices (online Supplementary Fig. S4). Depression severity showed the highest level of betweenness, closeness and strength. ADHD symptom severity showed high levels of betweenness and closeness. Anhedonia severity showed a high strength value. The CS-coefficients were 0.75 for strength, 0.59 for closeness, and 0.21 for betweenness. The values of strength and closeness are above the recommended threshold between 0.25 and 0.5, suggesting that these are the most stable centrality indices. The CS-coefficient for betweenness is below the recommended threshold and should thus be interpreted more cautiously (Epskamp et al., 2018). Results of the associated sample-dropping bootstrap are presented in online Supplementary Fig. S5. We tested whether nodes significantly differed in node strength (online Supplementary Fig. S6) and closeness (online Supplementary Fig. S7). As strength and closeness were the most stable centrality indices, we focused solely on them (Armour, Fried, Deserno, Tsai, & Pietrzak, 2017; Heeren, Bernstein, & McNally, 2018). Accordingly, depression severity and anhedonia severity were significantly more central than the other nodes.

#### Relative importance network

**Fig. 2.**
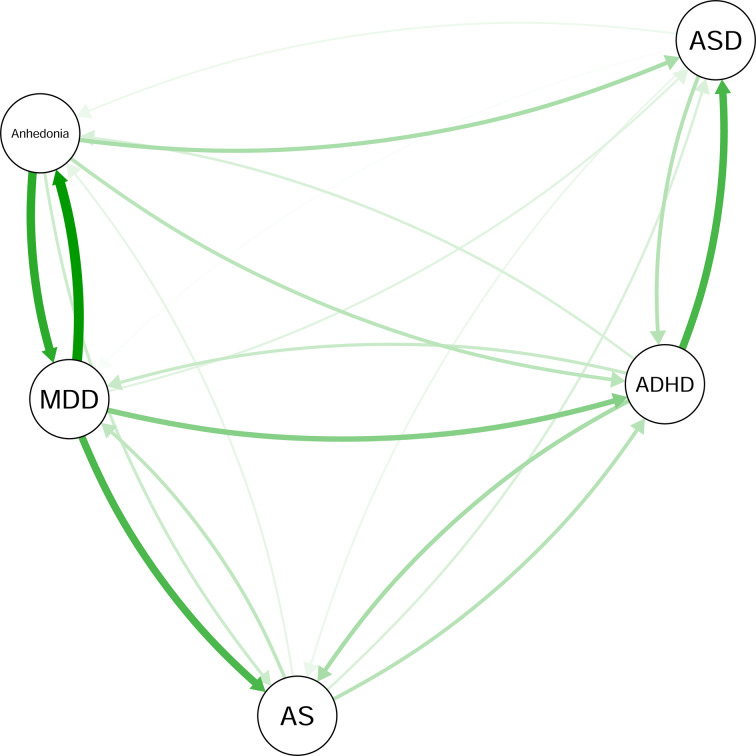
Relative importance network. Nodes represent anhedonia severity or severity of symptom clusters of MDD, AS, ADHD, or ASD. All edges reflect the relative importance of a node as a predictor of another node. Each thickness represents its magnitude. Arrows denote predictive directionality.

Figure 2 depicts the relative importance network. The relative importance values (i.e. *lmg*) are presented in online Supplementary Table S3. We found that depression severity was strongly predictive of anhedonia (*lmg* = 0.75), AS (*lmg* = 0.53) and ADHD (*lmg* = .36) symptom severity. Anhedonia was predictive of depression (*lmg* = 0.62), ASD (*lmg* = 0.25), ADHD (*lmg* = 0.20), and AS (*lmg* = .15) symptom severity. Although anhedonia strongly predicted AS (*lmg* = .15), AS predicted ADHD severity as well (*lmg* = 0.21), which in turn predicted anhedonia severity (*lmg* = 0.11). Moreover, ADHD symptom severity was strongly predictive of ASD symptom severity (*lmg* = 0.54). The bootstrapped CIs for the edges indicated again that the edges were fairly stable (online Supplementary Fig. S8).

#### Node centrality

**Fig. 3.**
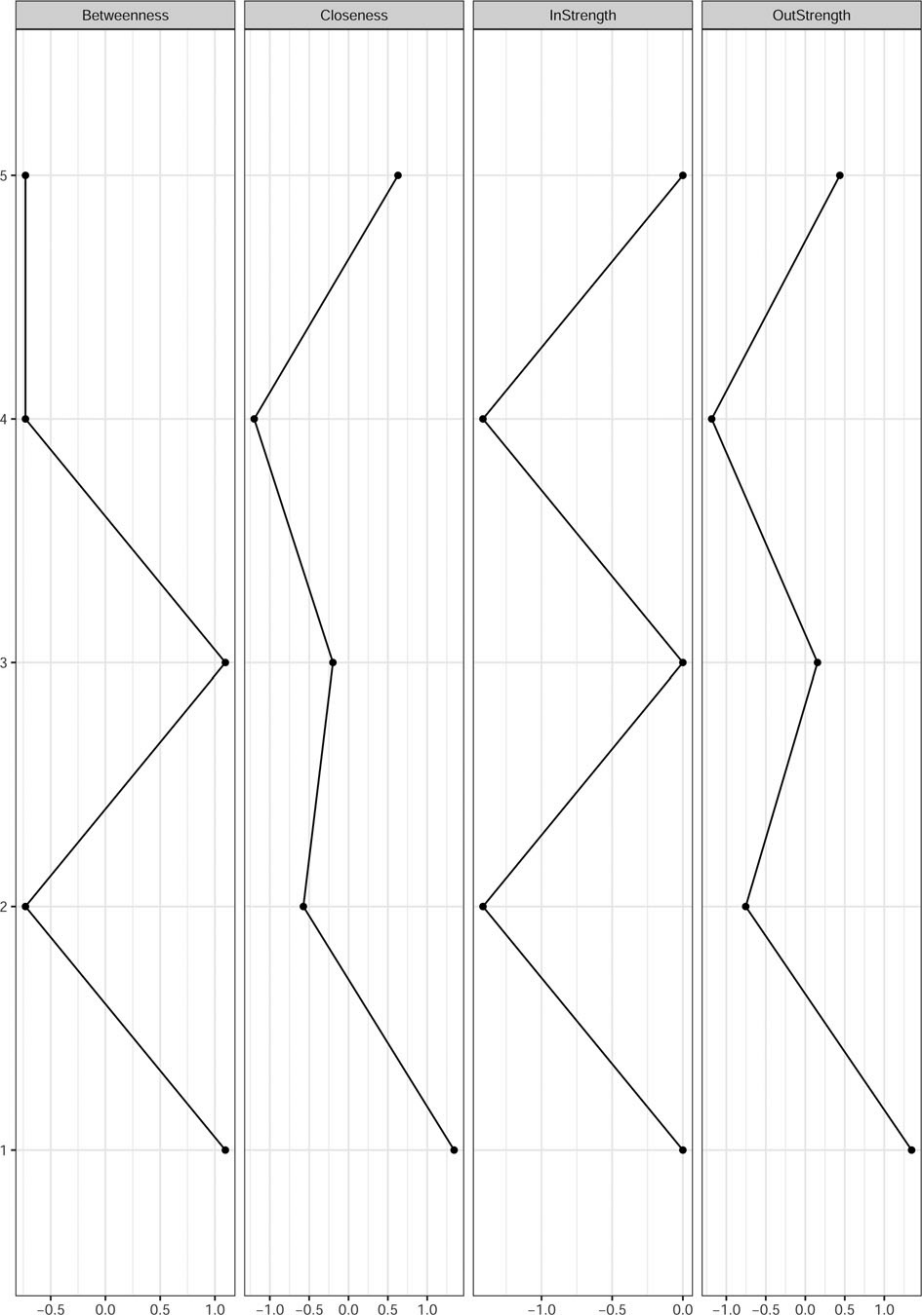
Centrality plot for the relative importance network depicting standardized measures of nodes’ betweenness, closeness, in-strength and out-strength. Nodes represent anhedonia severity or severity of symptom clusters of MDD, AS, ADHD, or ASD. More positive values indicate stronger associations with other nodes in the network.

These relationships are also reflected in the centrality indices (Fig. 3). Depression and ADHD symptom severity showed the highest levels of betweenness centrality. Depression and anhedonia severity showed the greatest closeness centrality. Depression and anhedonia severity yielded the highest out-strength. Depression severity, ADHD symptom severity and anhedonia severity demonstrated the highest in-strength values. The results of the associated case-dropping bootstrap suggest that all centrality indices were stable, except for in-strength. The CS-coefficients were 0.75 for out-strength, 0 for in-strength, 0.75 for closeness, and 0.59 for betweenness. As the CS-coefficient for in-strength is below the recommended cutoff between 0.25 and 0.5 (Epskamp et al., 2018), in-strength cannot be interpreted reliably.

#### Additional analyses

The anhedonia and depression severity nodes may be measuring overlapping constructs (i.e. anhedonia is a core symptom of MDD and hence also assessed in the IDS-SR). This could have inflated edge weights and centrality, which makes it difficult to ensure that the strong connection between the nodes is not due to their conceptual overlap (Rodebaugh et al., 2018). To control for this possibility, we computed an additional network without the overlapping anhedonia items in the IDS-SR. This resulted in a similar network structure (online Supplementary Fig. S9). Additionally, we tested an exploratory network without the MDD node, to determine more unique relationships between anhedonia and the other symptoms clusters independent of MDD symptomatology. The resulting network demonstrates stronger relationships between anhedonia and symptom clusters of AS, ADHD and ASD while the overall pattern of results was highly similar to the original model. We present these additional exploratory results in the Supplemental materials.

## Discussion

Mental disorders as defined by DSM classifications are highly comorbid (Antshel et al., 2016; Ghaziuddin et al., 2002; Grevet et al., 2006; Hirschfeld, 2001; Joshi et al., 2013; Kaufman & Charney, 2000; Kessler et al., 2006a, 2006b; Schatz & Rostain, 2006; van Loo et al., 2013). These high levels of comorbidity make it important to look for underlying constructs. In line with the RDoC framework, this study explored the role of anhedonia as an underlying feature associated with the severity of symptom clusters of depression, AS, ADHD, and ASD, by computing two types of networks. The most interesting results were the central role of depression severity and anhedonia severity within the network.

Anhedonia severity yielded a high strength centrality value in both networks. The relative importance network illustrated that anhedonia severity is predictive of the severity of symptom clusters of depression, AS, ASD, and ADHD, with the statistical prediction of depression severity being the strongest. Interestingly, the influence of anhedonia severity on these symptom clusters was stronger than the reverse influences on anhedonia severity. These results offer new insights into the ongoing discussion whether anhedonia is depression specific. In line with Watson and Naragon-Gainey (2010), we specifically found that anhedonia is more strongly predictive of depression than AS. Thus, anhedonia is not confined solely to depression, but also seems to characterize – to a lesser degree – AS and possibly anxiety disorders (Kashdan et al., 2008; Keedwell et al., 2005; Loas et al., 1992; Rey et al., 2009; Schrader, 1997; Vrieze et al., 2014a, 2014b). Moreover, the results suggest that anhedonia severity is predictive of and therefore potentially contributes to more severe ASD and ADHD symptom severity. This suggests that anhedonia may serve as a common underlying transdiagnostic psychopathology feature predictive of symptom severity of AS, ADHD, and ASD.

Although anhedonia severity was strongly predictive of more severe depression symptoms, depression symptom severity was slightly more predictive of anhedonia severity than vice versa. This suggests that more severe depression symptoms may foster anhedonia and that anhedonic individuals may be prone to experience more severe depression. This interplay between depression symptoms in general and anhedonia as a core depressive symptom might be a maintaining feature of depressive symptom severity. These relationships appear consistent with theory stating that anhedonia may be the most influential MDD symptom (Beard et al., 2016; Fried, Epskamp, Nesse, Tuerlinckx, & Borsboom, 2016; Rosenström et al., 2015) and that more severe depression could also result in more severe anhedonia (Beard et al., 2016). Interestingly, the central role of anhedonia corresponds to empirically based treatments for MDD. For example, behavioral activation, in which individuals are promoted to engage in positively reinforcing activities, may be particularly helpful for individuals who display anhedonia (Naragon-Gainey, Watson, & Markon, 2009; Walsh et al., 2019). Based on the central role of anhedonia in our analyses, behavioral activation may serve as an important initial treatment strategy for patients presenting with (comorbidity of) depression, anxiety disorders, ADHD, or ASD.

Depression severity was even more central in the network than anhedonia severity. Depression severity was predictive of more severe anhedonia, AS, ADHD, and ASD symptoms. Interestingly, these relationships were stronger than the reverse influences of the symptom clusters on depression severity. It is possible that more severe depression strengthens other present symptoms such as irritability, nervousness, feeling restless, and concentration problems (Beard et al., 2016; Meinzer et al., 2014; Park & Kim, 2020), leading to more severe AS, ADHD or ASD symptoms (Breslau & Davis, 1985; Bron et al., 2016; Loas et al., 1992).

It is also possible that some of the relationships between depression severity and the symptom clusters may be explained by the high betweenness value of ADHD symptom severity (Figs. 3 and S4). This betweenness value could only be interpreted in the relative importance network, and therefore these results should be interpreted with caution. If the results will be replicated in future studies, this value could suggest that the importance of ADHD symptom severity is derived from its central position in the network, rather than the strength of relationships. Accordingly, ADHD symptom severity may perhaps serve as a symptom cluster that contains both symptoms related to emotional disorders and neurodevelopmental disorders (Antshel et al., 2016). Previous studies already illustrated that MDD and ADHD, and ADHD and ASD are strongly associated, largely due to genetic factors (Ghirardi et al., 2019; Lee et al., 2013), but there might be additional symptom dimensions underlying the comorbidity between them. For example, cognitive biases that are thought to underlie vulnerability for MDD have also been found in individuals with increased ADHD symptoms (Lemoult & Gotlib, 2018; Vrijsen et al., 2018), whereas individuals with ASD only are not found to display these cognitive biases (Bergman et al., 2020). One possibility is that cognitive vulnerability factors (e.g. attentional difficulties, selective attention bias, or negative memory bias to all emotional cues; Antshel et al., 2016; Lawson et al., 2015) serve as a symptom cluster bridging symptoms related to MDD with those related to neurodevelopmental disorders, making ASD symptoms more pronounced. A relevant question for future studies is to gain more evidence for such an intermediate role of ADHD related symptom clusters and the potential clinical relevance for treating those cognitive vulnerability factors.

Interestingly, depression severity was more strongly predictive of AS and ADHD symptom severity than anhedonia severity. This suggests that another aspect of depression severity, other than the core symptom anhedonia, may be related to AS and ADHD symptom severity. Indeed, ADHD and anxiety symptoms are increased among individuals with more severe depression (Bron et al., 2016; Rector, Szacun-Shimizu, & Leybman, 2007). More studies that disentangle anhedonia and other depression symptoms are needed to verify whether other aspects of depression severity are related to AS and ADHD symptom severity. Thus, even though anhedonia seems to be a common underlying transdiagnostic psychopathology feature of depression, AS, ADHD, and ASD symptom severity, there are other emotional, (neuro-)cognitive, or biological factors at play driving high comorbidity between these symptom clusters and disorders.

Overall, our findings suggest that anhedonia may serve as a common underlying transdiagnostic psychopathology feature, associated with symptom severity of depression, AS, ADHD, and ASD (Chevallier et al., 2011; Loas et al., 1992; Meinzer et al., 2014; Novacek et al., 2016). On a biological level, this relationship may be explained by a dysfunction in the reward system and/or affect regulation (Chevallier et al., 2011; Keedwell et al., 2005; Krach et al., 2010; Meinzer et al., 2014; Pizzagalli et al., 2008; Vrieze et al., 2014a, 2014b). Future studies should therefore clarify the relation between dysfunctions in the reward system and/or affect regulation and symptom severity of depression, AS, ADHD, and ASD by using a different level of analysis as documented in the RDoC matrix (e.g. brain circuits or different research paradigms).

Strengths of this study include the use of a large clinical sample and the use of the network approach to investigate the transdiagnostic role of anhedonia related to the severity of symptom clusters, instead of a focus on separate diagnostic categories (Cuthbert, 2014; Insel et al., 2010). Moreover, we did not exclude participants with comorbid disorders. This approach is representative of clinical practice, where patients are often diagnosed with comorbid disorders that cannot be easily disentangled (Zbozinek et al., 2012). Finally, the use of a heterogeneous clinical sample provided variability among all symptoms, including symptoms outside of patients' diagnosed disorder(s).

Our study has several limitations as well. First, anhedonia was measured using the anhedonia factor structure of the OQ-45 (Minami et al., 2009), instead of a validated measure. Although the internal consistency of this factor was good (*α* = 0.87), its use limits the generalizability and perhaps the reliability and validity of our results because this is not validated. Hence, our results should be interpreted with caution and replication of these findings with validated anhedonia measures, such as the SHAPS (Snaith et al., 1995) or DARS (Rizvi et al., 2015) is warranted. However, at the time of the start of MIND-Set, no Dutch validated versions of these self-report measures were available. Second, our results cannot be generalized to non-help seeking patients and/or community-based samples, given that we used a clinical sample. In addition, it is possible that individuals with more severe symptoms are less likely to enter psychological treatment or that individuals with a milder form of psychopathology are too engaged in different activities to seek help. We unfortunately cannot evaluate whether this influenced the inclusion of patients in our study. However, the OQ-45 total score (*M* *=* 76.9, s.d. = 24.5) indicates that participants psychological distress is of clinical significance, based on the recommended Dutch cut-off (i.e. cut-off score = 55; De Jong, Nugter, Lambert, & Burlingame, 2009) and the American cut-off (i.e. cut-of score = 63; Lambert, Gregersen, & Burlingame, 2004). Although a selection bias might be less potent in the study compared to studies where non-clinical care data is used, a selection bias might have a strong influence on our results which we cannot fully address or rule out. Third, clinicians assessed all diagnoses using validated structured interviews (Luderer et al., 2020). Despite these high-standard efforts, we cannot completely rule out that ADHD is underdiagnosed, also given that ADHD is often underrecognized in patients with substance use disorders (Huntley et al., 2012). Moreover, the structured interviews did not provide information about which diagnosis was primary or secondary and information from the clinician about the presumed primary or secondary diagnosis was not included in our prospective study design and we cannot access such information retrospectively. However, the aim of our study was to explicitly look across diagnostic boundaries to explore transdiagnostic symptom profiles, instead of diagnoses. Thus, we present the diagnoses of participants to give an overview of sample characteristics but did not use this information in our statistical analyses. Instead, we focused on symptom clusters based on questionnaire scores. Therefore, a possible underdiagnosis of ADHD as well as a lack of information about primary/secondary diagnoses, would not have affected our results. Fourth, a small number of patients in each diagnostic group rendered subgroup analyses of ‘pure’ diagnostic categories without comorbidity (e.g. those with solely MDD), impossible. Future studies interested in disorder-specific contributions of anhedonia could investigate such networks on the symptom level within one diagnostic category. Finally, the network approach as used in the current study is cross-sectional and therefore, our data suggests, but cannot confirm causal connections among nodes. In response to this limitation, we employed a relative importance network. The directionality does allow us to suggest potential hypotheses for future research into causal relations. The findings that anhedonia is related to symptom severity of depression, AS, ADHD, and ASD are consistent with theory (Chevallier et al., 2011; Keedwell et al., 2005; Novacek et al., 2016; Rey et al., 2009; Schrader, 1997). Longitudinal data and/or experimental data are needed to clarify the temporal and causal structure of the relations found in the present study.

These limitations notwithstanding, this study offers unique insights on plausible relationships between anhedonia severity and severity of symptom clusters that merit further study. While our results are mostly hypothesis-generating, they suggest anhedonia as a common underlying transdiagnostic psychopathology feature, predictive of the severity of symptom clusters of depression, AS, ADHD, and ASD. Thus, anhedonia may be associated with the high comorbidity between these symptom clusters and disorders. Moreover, our results suggest that depression severity is predictive of the severity of symptom clusters of AS and ADHD. If our results will be replicated in future studies, it is recommended for clinicians to be more vigilant about screening for anhedonia and/or depression severity in individuals diagnosed with an anxiety disorder, ADHD and/or ASD. Future studies should investigate whether a focus on anhedonia, as a potential underlying transdiagnostic psychopathology feature associated with symptom severity of depression, AS, ADHD and ASD, is therapeutically efficacious.

## Footnotes

† The notes appear after the main text.

## Notes

1 At the time at data collection, the DSM-4 was still used, (i.e. OCD and PTSD are still listed as anxiety disorders), but the overview provided is in line with DSM-5 classifications.

2 The total OQ-45-2 consists of 45 items describing psychological problems (e.g. depression, anxiety, substance dependence) and how people function during social contacts in their daily life (e.g. interpersonal relationships, school/work functioning), but no other parts of the OQ-45-2 were used.

## Supplementary material

For supplementary material accompanying this paper visit https://doi.org/10.1017/S0033291722000575. click here to view supplementary material
