# Supplementary material for: Anhedonia as a transdiagnostic symptom across psychological disorders: a network approach
Source: Psychol Med. 2022 Mar 29;53(9):3908–19. doi: 10.1017/S0033291722000575 (PMC10317820; doi:10.1017/S0033291722000575)
Supplement: Supplementary file 1 [file S0033291722000575sup001.docx]

**Supplemental Material**

*Figure S1.* Graphical LASSO network after performing a non-linear transformation. This network does not differ from the original network as depicted in Figure 1.

Table S2

*Correlation matrix for the network. Raw correlations are presented above the diagonal. Below the diagonal are partial correlations after graphical lasso regularization.*

|  | IDS-SR | ASI | CAARS-SS | AQ-50 | Anhedonia |
| --- | --- | --- | --- | --- | --- |
| IDS-SR | - | .484 | .481 | .182 | .727 |
| ASI | .279 | - | .384 | .191 | .322 |
| CAARS-SS | .215 | .173 | - | .323 | .404 |
| AQ-50 | .000 | .035 | .211 | - | .249 |
| Anhedonia | .607 | .000 | .067 | .091 | - |

IDS-SR, Inventory of Depressive Symptomatology Self-Report; ASI, Anxiety Sensitivity Index; CAARS-SS, Conners’ Adult ADHD Rating Scale, Short Version; AQ-50, 50-item Autism Spectrum Quotient.

Table S3

*Relative importance matrix for the relative importance network. Rows represent independent variables, and the columns represent dependent variables.*

|  | IDS-SR | ASI | CAARS-SS | AQ-50 | Anhedonia |
| --- | --- | --- | --- | --- | --- |
| IDS-SR | - | .532 | .360 | .089 | .758 |
| ASI | .188 | - | .213 | .111 | .071 |
| CAARS-SS | .164 | .258 | - | .544 | .116 |
| AQ-50 | .017 | .056 | 221 | - | .053 |
| Anhedonia | .629 | .152 | .204 | .254 | - |

IDS-SR, Inventory of Depressive Symptomatology Self-Report; ASI, Anxiety Sensitivity Index; CAARS-SS, Conners’ Adult ADHD Rating Scale, Short Version; AQ-50, 50-item Autism Spectrum Quotient.

*Figure S2.* Bootstrapped confidence intervals of estimated edge weights for the graphical LASSO network. The edges are arranged such that the one with the highest edge-weight is at the top and the lowest at the bottom. The red line indicates the sample values and the gray are the 95% confidence intervals. All bootstrap CIs were small enough to give a fair amount of confidence as to their stability.

*Figure S3.* Bootstrapped difference tests (α = 0.05) between edge-weights that were non-zero estimated in the network. Black boxes represent edges that do differ significantly from one another, and gray boxes indicate edges that do not differ significantly from one another. The diagonal represents the strength of the edge weights, changing from light blue (weaker edge) to dark blue (stronger edge).

*Figure S4.* Centrality plots for the graphical LASSO network depicting standardized measures of node betweenness, closeness, and strength. Nodes represent anhedonia severity or severity of symptom clusters of MDD, AS, ADHD, or ASD. More positive values indicate stronger associations with other nodes in the network.

*Figure S5.* Average correlation between centrality indices in the bootstrapped samples and the original sample as a function of participants in the bootstrapped sample.

*Figure S6.* Bootstrapped difference tests (α = 0.05) between node strengths. Black boxes represent nodes that do differ significantly from one another, and gray boxes indicate nodes that do not differ significantly from one another. The diagonal represents the standardized node strength.

*Figure S7.* Bootstrapped difference tests (α = 0.05) between node closeness. Black boxes represent nodes that do differ significantly from one another and gray boxes indicate nodes that do not differ significantly from one another. The diagonal represents the standardized node closeness.

*Figure S8.* Bootstrapped confidence intervals of estimated edge weights for the relative importance network. The edges are arranged such that the one with the highest edge-weight is at the top and the lowest at the bottom. The red line indicates the sample values. The gray area indicates the 95% confidence intervals.

*Figure S9.* Graphical LASSO network without overlapping anhedonia items in the IDS-SR. This network does not differ from the original network as depicted in Figure 1.

*Figure S10.* Graphical LASSO network without the MDD symptom cluster.

Table S4

*Correlation matrix of partial correlations after graphical lasso regularization.*

|  | ASI | CAARS-SS | AQ-50 | Anhedonia |
| --- | --- | --- | --- | --- |
| ASI | - | - | - | - |
| CAARS-SS | .272 | - | - | - |
| AQ-50 | .050 | .225 | - | - |
| Anhedonia | .187 | .280 | .123 | - |

*Figure S11.* Centrality plots for the graphical LASSO network depicting standardized measures of node betweenness, closeness, and strength. Nodes represent anhedonia severity or severity of symptom clusters of AS, ADHD, or ASD. More positive values indicate stronger associations with other nodes in the network.

*Figure S12.* Relative importance network. Nodes represent anhedonia severity or severity of symptom clusters of AS, ADHD, or ASD. All edges reflect the relative importance of a node as a predictor of another node. Each thickness represents its magnitude. Arrows denote predictive directionality.

Table S5

*Relative importance matrix for the network without the MDD symptom cluster. Rows represent independent variables, and the columns represent dependent variables.*

|  | ASI | CAARS-SS | AQ-50 | Anhedonia |
| --- | --- | --- | --- | --- |
| ASI | - | .340 | .136 | .302 |
| CAARS-SS | .542 | - | .590 | .538 |
| AQ-50 | .094 | 254 | - | .158 |
| Anhedonia | .362 | .405 | .273 | - |

*Figure S13.* Centrality plot for the relative importance network depicting standardized measures of nodes’ betweenness, closeness, in-strength and out-strength. Nodes represent anhedonia severity or severity of symptom clusters of AS, ADHD, or ASD. More positive values indicate stronger associations with other nodes in the network.

**Results of network without MDD symptom cluster**

Anhedonia was connected to AS, ASD and ADHD symptom severity (Figure S10). The correlation matrix with all the regularized partial correlation coefficients is presented in Table S4. ADHD symptom severity and anhedonia severity showed the highest strength value and ADHD symptom severity showed the highest closeness value (Figure S11). The CS-coefficients were .594 for strength, .517 for closeness and .284 for betweenness.

Figure S12 depicts the relative importance network. The relative importance values are presented in table S5. Anhedonia was predictive of ADHD (*lmg* = .40), AS (*lmg* = .36) and ASD (*lmg* = .27) symptom severity. ADHD symptom severity was strongly predictive of AS (*lmg* = .54), ASD (*lmg* = .59) and anhedonia (*lmg* = .53) symptom severity. ADHD symptom severity and anhedonia severity showed the highest levels of out-strength. ADHD symptom severity showed the highest level of closeness (Figure S13). The CS-coefficients were .671 for out-strength, 0 for in-strength, .671 for closeness and .438 for betweenness.
